# Robotic‐Arm Assisted Direct Anterior Approach Total Hip Arthroplasty Facilitates Tensor Fasciae Latae Preservation and Early Functional Recovery in Obese Patients

**DOI:** 10.1111/os.70444

**Published:** 2026-10-12

**Authors:** Shijie Chen, Dasheng Lin, Huihuang Chen, Wentao Lin, Dongmin Xu, Eryou Feng

**Affiliations:** ^1^ Department of Orthopedics Fujian Medical University Union Hospital Fuzhou China

**Keywords:** direct anterior approach, functional recovery, muscle preservation, obesity, robotic‐arm assisted, total hip arthroplasty

## Abstract

**Objective:**

This study aimed to evaluate the efficacy and safety of robotic‐arm assisted (RA) direct anterior approach (DAA) total hip arthroplasty (THA) in facilitating tensor fasciae latae (TFL) preservation and subsequent functional recovery, and to explore the correlation between TFL preservation and postoperative inflammation, pain, and early functional recovery in obese patients.

**Methods:**

We retrospectively reviewed 69 obese patients (BMI ≥ 30 kg/m^2^) undergoing primary DAA‐THA from September 2023 to December 2024 with 12‐month follow‐up. Surgical procedures were performed by RA technology in 31 patients (RA group) and manual technology in 38 patients (M group). Demographic characteristics, perioperative parameters, TFL injury index, unsupported ambulation duration, creatine kinase (CK), C‐reactive protein (CRP), Visual Analog Scale scores (VASs), Harris Hip Scores (HHS), Western Ontario and McMaster Universities Osteoarthritis Index (WOMAC), Forgotten Joint Score‐12 (FJS‐12), radiologic measurements, and complications were retrieved from patients. Student's *t*‐test and Mann–Whitney *U* test were used to compare continuous data. Fisher test and Chi‐square test were used to compare the categorical data. Longitudinal data were evaluated by generalized estimating equations. The relationships between TFL injury index and early clinical outcomes were evaluated using Spearman's correlation analysis.

**Results:**

Baseline demographic characteristics of both groups were comparable. RA group demonstrated significantly lower TFL injury index (18.05 [IQR 14.51–30.05] % vs. 38.31 [IQR 20.58–50.70] %, *p* = 0.001) and shorter unsupported ambulation duration (3.00 [IQR 2.00–4.00] week vs. 5.00 [IQR 3.00–6.00] week, *p* < 0.001) compared to M group. Besides, TFL injury index showed a strong positive correlation with unsupported ambulation duration (*ρ* = 0.775) (*p* < 0.001). Compared to M group, RA group significantly reduced CK, CRP, and VASs at the initial 72 h postoperatively (all *p* < 0.05). RA group exhibited significantly superior HHS, WOMAC, and FJS‐12 compared to M group at postoperative 1 month (all *p* < 0.01). TFL injury index demonstrated strong positive correlations with CK (*ρ* = 0.619), CRP (*ρ* = 0.628), and VASs (*ρ* = 0.652) at postoperative 72 h (all *p* < 0.001). Furthermore, it showed a strong negative correlation with HHS (*ρ* = −0.615) and FJS‐12 (*ρ* = −0.759), and moderate positive correlations with WOMAC‐pain (*ρ* = 0.410) and WOMAC‐function (*ρ* = 0.583) at postoperative 1 month (all *p* < 0.001). Despite longer operative duration in RA group, it exhibited a significantly higher proportion of acetabular components positioned within the Lewinnek safe zone and a significantly lower incidence of incision‐related adverse events compared to M group (all *p* < 0.05).

**Conclusion:**

In obese patients undergoing DAA‐THA, RA technology significantly reduced intraoperative injury to TFL. This muscle preservation effect was significantly associated with attenuated early postoperative inflammation, lower pain levels, and faster functional recovery.

## Graphical abstract

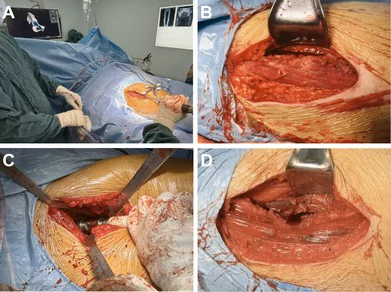

**Abbreviations**
3D: three‐dimensional
AO: acetabular offset
ASA: American Society of Anesthesiologists
BMI: body mass index
CT: computed tomography
DAA: direct anterior approach
FJS‐12: Forgotten Joint Score‐12
FO: femoral offset
GEEs: generalized estimating equations
GO: global offset
HHS: Harris Hip Score
ICC: intraclass correlation coefficient
LLD: limb length discrepancy
MRI: magnetic resonance imaging
Post‐op 12mo: postoperative 12 months
Post‐op 1mo: postoperative 1 month
Post‐op 24h: postoperative 24 h
Post‐op 6mo: postoperative 6 months
Post‐op 72h: postoperative 72 h
Pre‐op: preoperative
PROMs: patient‐reported outcome measures
QIC: quasi‐likelihood under the independence model criterion
RA: robotic‐arm assisted
TFL: tensor fasciae latae
THA: total hip arthroplasty
VAS: Visual Analog Scale
WOMAC: Western Ontario and McMaster Universities Osteoarthritis Index
Δ: mean difference

## 1 Introduction

Direct anterior approach (DAA) total hip arthroplasty (THA) utilizes an intermuscular interval for joint access, preserving muscular integrity without requiring myotomy which confers advantages including minimal surgical trauma, physiological gait restoration, reduced dislocation risk, and accelerated functional recovery [1, 2, 3, 4]. However, increased soft‐tissue envelope thickness impairs surgical exposure and restricts instrument maneuverability in obese patients undergoing DAA‐THA. When performing DAA‐THA in this population, more extensive dissection around the acetabulum and proximal femur is required, while surgical instruments exert greater tension and shear forces on the periarticular soft tissues. Consequently, these factors collectively increase susceptibility to postoperative wound complications in obese patients, including surgical site infection, dehiscence, and persistent incisional pain [5, 6, 7, 8, 9]. Furthermore, these anatomical constraints also elevate the technical complexity of achieving precise components positioning [10, 11, 12, 13]. Contemporary evidence further associated DAA with tensor fasciae latae (TFL) injury, heightened analgesic requirements, prolonged hospitalization, and compromised early‐phase rehabilitation in obese patients [5, 14, 15]. Collectively, these limitations pose unique challenges for implementing DAA‐THA in obese patients.

Robotic‐arm assisted (RA) technology utilizes three‐dimensional (3D) planning based on preoperative computed tomography (CT) to guide high‐precision bone resection and accurate implant positioning during THA, thereby reducing human error [16, 17]. It is widely accepted that high‐precision surgical execution effectively mitigates periarticular soft‐tissue injury during THA, while reducing postoperative pain and muscular dysfunction [18, 19]. More importantly, the robotic arm incorporates real‐time stereotactic feedback with haptic boundaries that confines surgical instrumentation within the preoperatively surgical plan. This constraint mitigates excessive soft tissue tension and shear forces, reducing iatrogenic injury [20]. However, the impact of robotic‐arm assisted DAA‐THA on the periarticular muscles and subsequent functional recovery in obese patients remains inadequately investigated.

Given the rising global obesity burden, optimizing THA outcomes in obese patients is critical. However, the impact of RA technology on musculature and early functional recovery in this population remains undocumented in the extant literature. To address this knowledge gap, we designed this retrospective controlled cohort study with the dual objectives of: (i) Evaluating the efficacy and safety profile of robotic‐arm assisted DAA‐THA for enhancing TFL preservation and subsequent functional recovery in obese population. (ii) Exploring the correlation between TFL preservation and postoperative inflammation, pain, and early functional recovery to furnish evidence‐based support for improving arthroplasty outcomes in obese population.

## 2 Patients and Methods

### 2.1 Study Design

This single‐center retrospective cohort study was conducted in accordance with the Declaration of Helsinki while approved and supervised by the Ethics Committee of our institution (Approval No. 2025KY408), which provided continuous oversight throughout the investigation period. The Ethics Committee granted a waiver of consent for this retrospective study.

### 2.2 Patients

We reviewed clinical records and radiographic data from obese patients undergoing primary DAA‐THA utilizing RA technology (RA group) or manual technology (M group) at our institution from September 2023 to December 2024.

Inclusion criteria comprised: (i) End‐stage hip pathology necessitating arthroplasty, including osteochondrosis of femoral head (Ficat stages III–IV), developmental dysplasia of hip (acetabular anteversion < 40°, Crowe types I–II), hip osteoarthritis, rheumatoid arthritis, or ankylosing spondylitis; (ii) primary THA procedure; (iii) body mass index (BMI) ≥ 30 kg/m^2^.

Exclusion criteria comprised: (i) Revision arthroplasty cases; (ii) severe developmental dysplasia of hip (Crowe types III–IV); (iii) comorbid conditions potentially compromising postoperative rehabilitation, including advanced knee arthropathy, cerebrovascular disease, functionally significant neurological disease, or psychiatric disorders.

A total of 92 patients were reviewed, of whom 23 did not meet the inclusion criteria, 19 refused to participate, and 4 dropped out for missing follow‐up. Finally, 69 patients completed the minimum 12‐month follow‐up, comprising 31 in RA group and 38 in M group (Figure 1). Baseline demographic characteristics of both groups were comparable (Table 1).

**FIGURE 1:**
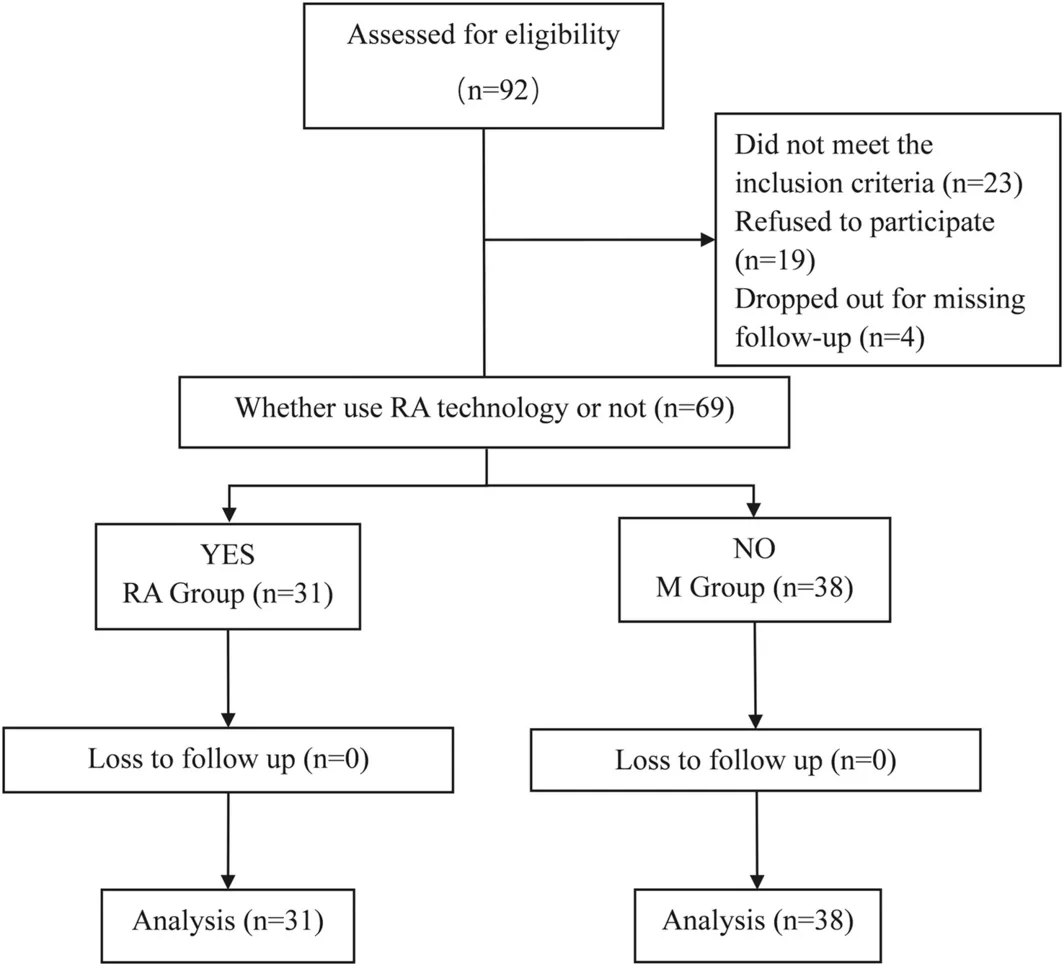
Flowchart of recruiting participants and analysis.

**TABLE 1:** Baseline demographic characteristics of both groups.

	RA group (*n* = 31)	M group (*n* = 38)	Statistic values	*p*
Age (years)	68.00 (IQR 56.00**–**73.00)	62.37 ± 12.02	*Z* = −0.773	0.440
Gender			*χ* ^2^ = 0.004	0.948
Men	12 (38.7%)	15 (39.5%)		
Women	19 (61.3%)	23 (60.5%)		
BMI (kg/m^2^)	31.30 (IQR 31.10–31.73)	31.63 (IQR 31.11–32.30)	*Z* = −1.279	0.201
ASA classification (*n*)			*χ* ^2^ = 1.208	0.272
II	19 (61.3%)	28 (73.7%)		
III	12 (38.7%)	10 (26.3%)		
Preoperative diagnosis (*n*)			*χ* ^*2*^ = 3.910	0.427
Hip osteoarthritis	3 (9.7%)	5 (13.2%)		
Osteochondrosis of femoral head	21 (67.7%)	17 (44.7%)		
Developmental dysplasia of hip	5 (16.1%)	12 (31.6%)		
Ankylosing spondylitis	1 (3.2%)	2 (5.3%)		
Others	1 (3.2%)	2 (5.3%)		
Preoperative complications (*n*)			*χ* ^2^ = 8.811	0.249
Hypertension	8 (25.8%)	15 (39.5%)		
Diabetes	5 (16.1%)	2 (5.3%)		
Coronary heart disease	3 (9.7%)	1 (2.6%)		
Cardiac insufficiency	2 (6.5%)	1 (2.6%)		
Renal insufficiency	1 (3.2%)	3 (7.9%)		
Hepatic insufficiency	2 (6.5%)	2 (5.3%)		
Connective tissue disease	0 (0.0%)	4 (10.5%)		
None	10 (32.3%)	10 (26.3%)		

### 2.3 Surgery

All surgical procedures were performed by a single senior orthopedic surgeon (EF) who had surpassed the learning curve for robotic‐arm assisted and manual DAA‐THA prior to this study period, ensuring operational proficiency that minimized technique‐related confounders [21, 22]. Patients in both groups underwent implantation with the Trident acetabular cup and the Accolade II femoral stem (Stryker Orthopedics, Mahwah, NJ, USA). General anesthesia with lumbar plexus block was administered by a dedicated anesthesiology team for all cases.

The DAA‐THA were performed following a standardized protocol which prioritized stability and biomechanical restoration of hip [23, 24, 25]. A 10 to 15 cm incision was initiated 1.5 cm distal and lateral to the anterior superior iliac spine, oriented toward the fibular head. Surgical dissection proceeded through the intermuscular interval between the TFL and sartorius muscles, with deep exposure extending lateral to the rectus femoris. Ligation of ascending branches of the lateral femoral circumflex vessels was performed under direct visualization. Anterolateral capsulotomy was executed in a U‐shaped configuration along the rectus femoris‐gluteus minimus interval.

In M group, we utilized the conventional manual technique to perform femoral neck osteotomy 1 cm superior to the lesser trochanter, followed by femoral head extraction. After acetabular exposure with labral resection and circumferential soft‐tissue debridement, sequential acetabular reaming was performed until achieving concentric bony contact. The definitive acetabular prosthesis was implanted at 40° abduction and 15° anteversion. Following confirmation of satisfactory press‐fit stability, supplemental fixation with 1 to 2 screws was applied when indicated, and the corresponding liner was seated. The operative limb was then placed in traction to facilitate exposure and release of proximal femoral soft tissues. With the operating table flexed 30° downward, the hip was positioned in hyperextension, adduction, and external rotation to expose the proximal femoral canal. Femoral preparation commenced with a box osteotome at the calcar, followed by sequential broaching until achieving optimal cortical contact and rotational stability. The appropriately sized femoral stem and head prosthesis were then implanted.

In RA group, we utilized the Mako Robotic‐Arm Assisted Total Hip system (Stryker Orthopedics, Mahwah, NJ, USA) with THA 3.1 software for CT‐based planning, real‐time haptic‐guided acetabular preparation and impaction, limb length optimization, and offset restoration [18]. In brief, the procedural workflow initiated with the insertion of two bone screws into the contralateral anterior superior iliac spine to secure the navigation reference array, complemented by electrode fixation at the inferior patellar pole for intraoperative limb length assessment. Following initial exposure, fiducial markers were inserted at the acetabular rim and greater trochanter to establish baseline limb length and offset parameters. Pelvic registration incorporated three acetabular orientation points, 32 fiducial markers, and eight verification points, enabling automated co‐registration of preoperative 3D reconstructions with intraoperative anatomy via surface matching algorithms. After co‐registration, surgical execution relied entirely on the real‐time visual cues from the console and the constrained feedback of the robotic arm. The use of retractors was limited to critical steps requiring enhanced exposure (such as femoral head removal, instrument changes, reaming, and implant placement), thereby enabling the procedure to be performed predominantly without direct visualization. Acetabular reaming and prosthesis implantation were executed under robotic‐arm guidance with haptic constraints, ensuring adherence to virtual planning parameters. Subsequent femoral preparation proceeded conventionally through proximal exposure, canal opening, broaching, and trialing. During trial reduction, dynamic limb length and offset data were acquired in real‐time by probing the pelvic array, greater trochanter fiducial, and patellar electrode. The system provided continuous intraoperative feedback via a graphical interface displaying millimeter‐level changes in limb length and offset parameters.

Intraoperative fluoroscopic verification of components positioning was routinely performed using a mobile C‐arm system. Final component implantation proceeded only after objective verification of stability, limb length equality, and offset restoration in both groups. Both groups underwent standardized anterior capsulorrhaphy followed by layered fascial closure with absorbable sutures while no surgical drains were placed prior to wound closure.

### 2.4 Perioperative Management

Identical standardized perioperative protocols encompassing multimodal analgesia, transfusion, thromboprophylaxis, antimicrobial prophylaxis, and rehabilitation were applied to both groups [26, 27]. Standardized rehabilitation commenced immediately post‐emergence from anesthesia, featuring isometric quadriceps contractions and ankle pump exercises. Ambulation was initiated on postoperative day 1 utilizing progressive assistive devices transitioning until unassisted ambulation was tolerated. Patients were discharged upon achieving functional independence in activities of daily living, adequate pain control with oral analgesics, and medical stability.

### 2.5 Clinical Evaluation

Perioperative metrics encompassed operative duration, perioperative blood loss, allogeneic transfusion, and duration of hospitalization were reviewed. After final component implantation, standardized intraoperative photographs of TFL were acquired using a Canon EOS 760D fitted with a Canon EF 100 mm f/2.8 IS USM lens (Canon Inc., Ohta‐ku, Tokyo, Japan). Photographs were obtained with the hip in a neutral position and TFL adequately exposed. The camera was mounted on a tripod positioned so that the lens axis was directed at the surgical field with the front element approximately 1 m from the tissue reference plane while preserving sterility, yielding an approximately 200 mm field width at that distance. Because the lens is a fixed 100 mm focal‐length macro design, no zoom adjustment was required. The aperture was set to f/8 with manual focus mode. Focus was confirmed on the principal tissue plane under live view and then locked for the session. The lens image stabilizer was turned off because the system was rigidly mounted. The camera was operated in full manual exposure at ISO 400 and 1/125 s. White balance was fixed using a custom preset measured under the operative lights. Exposures were triggered via a 2 s self‐timer. A sterile millimeter scale was placed within the same focal plane at the field margin to provide spatial calibration. Final figures were presented with scale bars derived from this reference. Any global adjustments were applied identically across images within both groups. Two independent investigators who were blinded to surgical techniques then performed retrospective, computer‐assisted morphometric analyses on these photographs using ImageJ software (NIH, USA). The maximum injury depth (*D*) and muscle belly width (*W*) were measured at the injury site, with measurement axes perpendicular to the fibers of TFL. The TFL injury index was defined as the ratio of the maximum injury depth and muscle belly width (*D*/*W*, %) (Figure 2). Measurements were performed in duplicate with a 2‐week washout period between sessions. The average of all measurement results was used for analysis to reduce bias and enhance precision. Creatine kinase (CK), C‐reactive protein (CRP), and Visual Analog Scale scores (VASs) during ambulation were assessed at preoperative, postoperative 24 h (Post‐op 24 h), and postoperative 72 h (Post‐op 72 h). Standardized clinical evaluations were conducted at postoperative 1 month (Post‐op 1mo), postoperative 6 months (Post‐op 6mo), and postoperative 12 months (Post‐op 12mo) documenting Harris Hip Scores (HHS), Western Ontario and McMaster Universities Osteoarthritis Index (WOMAC), Forgotten Joint Score‐12 (FJS‐12), unsupported ambulation duration, and postoperative complications (including incision pain, incision edema, abnormal healing, surgical site infection, deep venous thrombosis, periprosthetic joint infection, dislocation, aseptic loosening, reoperations, and readmissions).

**FIGURE 2:**
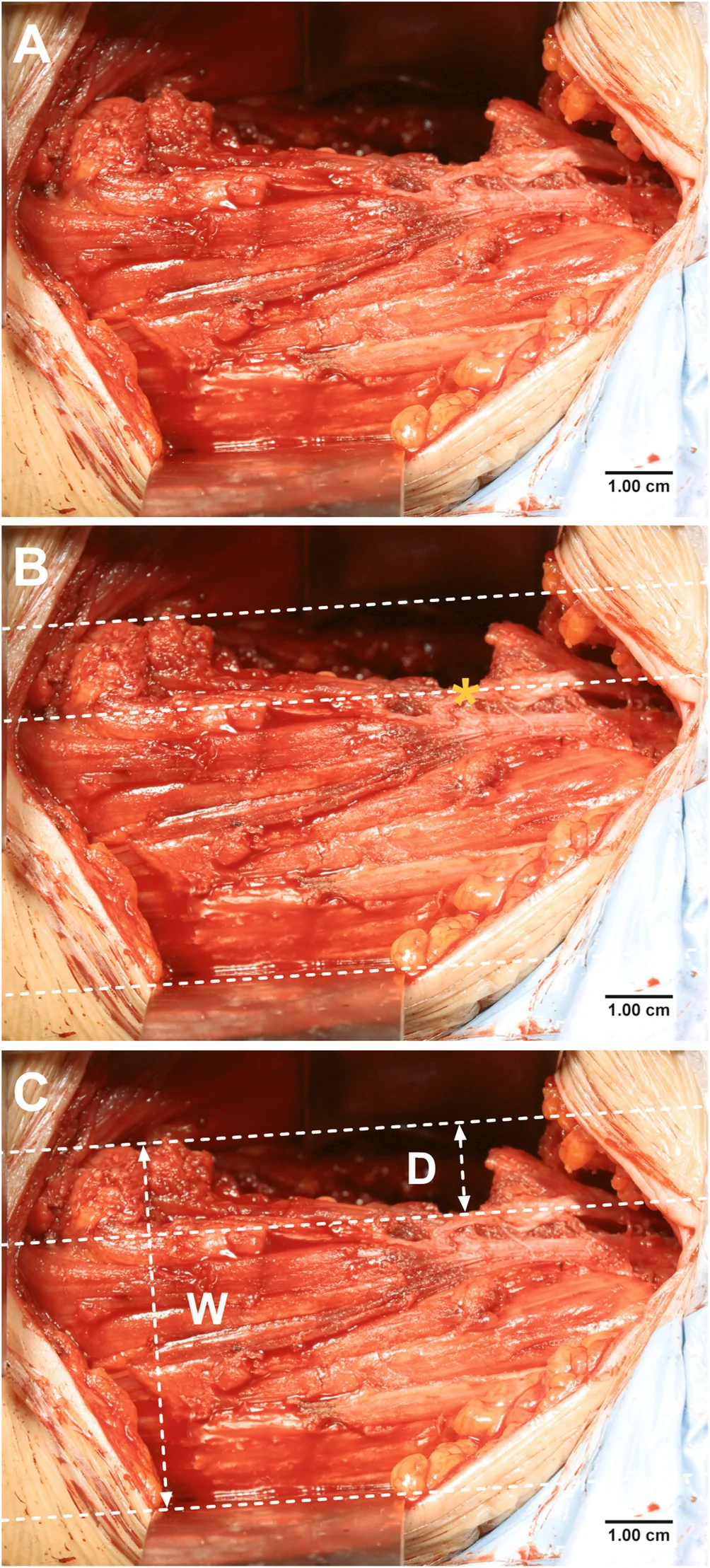
TFL injury in both groups was measured with TFL injury index. (A) Standardized intraoperative photographs of TFL were obtained using a programmed photographic protocol. (B) Three lines were drawn parallel to the fibers of TFL, passing through the superior and inferior borders of the muscle belly and the deepest point of the injury site. *The deepest point of the injury site. (C) The maximum injury depth (*D*) and muscle belly width (*W*) were measured perpendicular to the established parallel lines, and TFL injury index was calculated as *D*/*W* (%).

### 2.6 Radiologic Measurements

Radiologic measurements were independently performed by two independent investigators. The mean values of two measurements were calculated to mitigate bias and enhance precision. Acetabular abduction and anteversion angles were measured on standardized anteroposterior pelvic radiographs according to the methodologies described by Nho et al. [28] and Lewinnek et al. [29]. Acetabular prosthesis positioning within the Lewinnek safe zone (defined as 40° ± 10° abduction and 15° ± 10° anteversion) was documented. Limb length discrepancy (LLD) was quantified as the vertical distance difference from the lesser trochanter tip to the inter‐teardrop line, with positive values indicating operative limb lengthening. Femoral offset (FO) was measured from the femoral head center to the femoral anatomical axis. Acetabular offset (AO) represented the horizontal distance from the hip center to the midline sacrococcygeal junction. Global offset (GO) was defined as the sum of FO and AO. Offset discrepancies (FOD, AOD, and GOD) were calculated as operative minus contralateral values.

### 2.7 Statistical Analysis

All statistical analyses were performed using IBM SPSS Statistics version 23.0 (IBM Corp., Armonk, NY, USA). All statistics were based on two‐sided tests with the level of significance set at 0.05. Normally distributed data were expressed as mean and standard deviation. Skewed distributed data were expressed as median and interquartile range (IQR). Categorical data were expressed as frequencies and percentages. The Student's *t*‐test was used to compare normally distributed data, while the Mann–Whitney *U* test was used to compare skewed distributed data. Fisher's test and Chi‐square test were used to compare the categorical data. Longitudinal data were evaluated by Generalized Estimating Equations (GEEs) with Bonferroni correction for multiple comparisons. The relationships between TFL injury index and early clinical outcomes were evaluated using Spearman's correlation analysis.

The *E*‐value was calculated to quantify the robustness of TFL injury index to potential unmeasured confounding [30]. A larger *E*‐value indicates that stronger unmeasured confounding would be required to negate the observed effect, thereby supporting the robustness of the findings. Inter‐rater reliability of TFL injury index was assessed using a two‐way random‐effects model intraclass correlation coefficient (ICC) for absolute agreement, ICC (2,1). Intra‐rater reliability was evaluated using a two‐way mixed‐effects model ICC for consistency, ICC (3,1). To examine the criterion validity of TFL injury index, its association with postoperative serum CK levels was analyzed using Spearman's correlation analysis.

## 3 Results

### 3.1 Perioperative Parameters and Clinical Outcomes

No significant intergroup differences in perioperative blood loss, allogeneic transfusion, and duration of hospitalization (all *p* > 0.05). Compared to M group, RA group had a significantly longer operative duration (164.39 ± 55.07 min vs. 138.08 ± 53.51 min, *p* = 0.049) but a shorter unsupported ambulation duration (3.00 [IQR 2.00–4.00] week vs. 5.00 [IQR 3.00–6.00] week, *p* < 0.001).

RA group had significantly lower TFL injury index (18.05 [IQR 14.51–30.05] % vs. 38.31 [IQR 20.58–50.70] %, *p* = 0.001) compared to M group, representing a median difference of 20.26% (Table 2). The corresponding Cohen's *d* was 1.05, which, when converted to the OR scale using the Cox transformation, yielded an OR of 6.90 (95% CI: 2.75–17.30). The *E*‐value for this estimate was 14.3 (95% CI: 5.3–37.1). This means that an unmeasured confounder would need to be associated with both the exposure and the outcome by a risk ratio of at least 14.3‐fold each to fully explain away the observed association. Even the lower confidence limit (*E*‐value = 5.3) indicates that a strong unmeasured confounder would still be required. These results provide strong reassurance regarding the validity of our conclusions.

**TABLE 2:** Perioperative parameters and clinical outcomes of both groups.

	RA group (*n* = 31)	M group (*n* = 38)	Statistic values	*p*
Operative duration (min)	164.39 ± 55.07	138.08 ± 53.51	*t* = −2.005	0.049*
Duration of hospitalization (days)	4.00 (IQR 3.00–6.00)	4.50 (IQR 3.00–6.00)	*Z* = −0.031	0.976
Perioperative blood loss (mL)	662.88 (IQR 587.75–870.79)	855.43 ± 339.75	*Z* = −1.785	0.074
Allogeneic transfusion (*n*)				1.000^†^
Yes	0 (0.0%)	1 (2.6%)		
No	31 (100.0%)	37 (97.4%)		
TFL injury index (%)	18.05 (IQR 14.51–30.05)	38.31 (IQR 20.58–50.70)	*Z* = −3.221	0.001**
Unsupported ambulation duration (week)	3.00 (IQR 2.00–4.00)	5.00 (IQR 3.00–6.00)	*Z* = −4.120	< 0.001***

* *p* < 0.05.

** *p* < 0.01.

*** *p* < 0.001.

^†^ Fisher's exact test.

TFL injury index demonstrated excellent inter‐rater reliability, with ICC (2,1) of 0.819 (95% CI: 0.723–0.884, *p* < 0.001) and 0.793 (95% CI: 0.686–0.867, *p* < 0.001), respectively. The intra‐rater reliability for two investigators was also excellent, with ICC (3,1) of 0.820 (95% CI: 0.725–0.885, *p* < 0.001) and 0.780 (95% CI: 0.667–0.858, *p* < 0.001), respectively. Furthermore, Spearman's correlation analysis revealed strong positive correlations between TFL injury index and CK levels at Post‐op 24 h (*ρ* = 0.705) and Post‐op 72 h (*ρ* = 0.619) (all *p* < 0.001), supporting the criterion validity of this metric as an effective indicator of TFL injury (Table 4).

### 3.2 Serological Indicators and Patient‐Reported Outcome Measures

The GEEs detected significant group × time interaction effects in CK (Wald *χ* ^2^ = 21.699, *p* < 0.001), CRP (Wald *χ* ^2^ = 10.162, *p* = 0.006), VASs (Wald *χ* ^2^ = 7.275, *p* = 0.026), HHS (Wald *χ* ^2^ = 17.198, *p* = 0.001), WOMAC‐pain (Wald *χ* ^2^ = 8.641, *p* = 0.034), WOMAC‐function (Wald *χ* ^2^ = 9.067, *p* = 0.028), WOMAC‐stiffness (Wald *χ* ^2^ = 13.509, *p* = 0.004), and FJS‐12 (Wald *χ* ^2^ = 39.251, *p* < 0.001). Therefore, simple effects analyses were conducted to examine the effects of group and time separately. The QIC values for CK (QIC = 15,751,395.662), CRP (QIC = 154,699.080), VASs (QIC = 225.910), HHS (QIC = 15,972.132), WOMAC‐pain (QIC = 1787.443), WOMAC‐function (QIC = 6922.529), WOMAC‐stiffness (QIC = 371.288), and FJS‐12 (QIC = 21,131.132) reached their minima, indicating optimal correlation structure selection.

Simple effects analysis of temporal trends revealed: (i) Both groups demonstrated significantly higher CK and VASs at Post‐op 24 h than those at both preoperative and Post‐op 72 h (all *p* < 0.05). Additionally, CK levels at Post‐op 72 h remained elevated compared to preoperative baseline in both groups (both *p* < 0.001). However, no significant differences of VASs existed between preoperative baseline and Post‐op 72 h in either group (both *p* > 0.05) (Table 3, Figure 3A,B). (ii) Postoperative CRP levels increased progressively over time in both groups (all *p* < 0.001) (Table 3, Figure 3C). (iii) For within‐group longitudinal comparisons, both groups demonstrated statistically significant improvements and exhibited progressive enhancement over time in HHS, WOMAC‐pain, WOMAC‐function, and FJS‐12 (all *p* < 0.05). The WOMAC‐stiffness improved progressively in M group (all *p* < 0.01). In RA group, while a significant improvement in WOMAC‐stiffness was noted at Post‐op 1mo (*p* < 0.001), further changes in the scores at Post‐op 6mo and Post‐op 12mo were nonsignificant (all *p* > 0.05) (Table 3, Figure 3D–H).

**TABLE 3:** Simple effects analysis: Group × Time interaction for CK, CRP, VASs, HHS, WOMAC, and FJS‐12.

	RA group (*n* = 31)	M group (*n* = 38)	Δ (RA group − M group)^#^	*p* ^†^
CK (U/L)				
Pre‐op	79.00 (IQR 58.00–106.00)	73.50 (IQR 54.50–108.25)	5.63 (95% CI: −11.30 to 22.56)	0.514
Post‐op 24 h	619.00 (IQR 509.00–810.00)^‡^	954.50 (IQR 687.00–1195.50)^‡^	−300.81 (95% CI: −472.53 to −129.09)	0.001**
Post‐op 72 h	508.00 (IQR 334.00–667.00)^‡^ ^§^	818.50 (IQR 612.25–948.75)^‡^ ^§^	−295.78 (95% CI: −428.45 to −163.11)	< 0.001***
CRP (mg/L)				
Pre‐op	1.42 (IQR 0.72–3.06)	2.92 (IQR 1.18–5.86)	−1.00 (95% CI: −3.71 to 1.72)	0.472
Post‐op 24 h	19.79 (IQR 11.22–37.25)^‡^	41.32 (IQR 28.05–56.31)^‡^	−15.71 (95% CI: −26.49 to −4.93)	0.004**
Post‐op 72 h	78.53 (IQR 52.62–111.91)^‡^ ^§^	106.56 (IQR 76.34–134.69)^‡^ ^§^	−22.25 (95% CI: −41.91 to −2.59)	0.027*
VASs				
Pre‐op	5.00 (IQR 4.00–6.00)	5.00 (IQR 4.75–6.00)	−0.18 (95% CI: −0.64 to 0.27)	0.435
Post‐op 24 h	5.00 (IQR 5.00–6.00)^‡^	6.00 (IQR 6.00–7.00)^‡^	−0.86 (95% CI: −1.33 to −0.38)	< 0.001***
Post‐op 72 h	4.00 (IQR 3.00–5.00)^§^	5.50 (IQR 5.00–6.00)^§^	−1.12 (95% CI: −1.65 to −0.60)	< 0.001***
HHS				
Pre‐op	40.06 ± 10.11	42.63 ± 13.08	−2.57 (95% CI: −7.96 to 2.83)	0.351
Post‐op 1mo	70.58 ± 5.28^‡^	64.97 ± 8.67^‡^	5.61 (95% CI: 2.33 to 8.88)	0.001**
Post‐op 6mo	82.29 ± 5.72^‡^ ^§^	82.08 ± 5.05^‡^ ^§^	0.21 (95% CI: −2.32 to 2.75)	0.870
Post‐op 12mo	87.94 ± 4.67^‡^ ^§^ ^¶^	88.68 ± 3.15^‡^ ^§^ ^¶^	−0.75 (95% CI: −2.64 to 1.15)	0.439
WOMAC‐pain				
Pre‐op	12.29 ± 2.77	12.66 ± 2.94	−0.37 (95% CI: −1.70 to 0.96)	0.589
Post‐op 1mo	8.00 (IQR 7.00–11.00)^‡^	11.00 (IQR 7.00–13.25)^‡^	−2.16 (95% CI: −3.55 to −0.76)	0.002**
Post‐op 6mo	6.00 (IQR 4.00–8.00)^‡^ ^§^	6.00 (IQR 5.00–8.00)^‡^ ^§^	0.06 (95% CI: −0.98 to 1.10)	0.915
Post‐op 12mo	2.00 (IQR 2.00–5.00)^‡^ ^§^ ^¶^	3.50 (IQR 2.00–5.00)^‡^ ^§^ ^¶^	−0.61 (95% CI: −1.50 to 0.29)	0.184
WOMAC‐function				
Pre‐op	25.90 ± 9.02	26.34 ± 8.11	−0.44 (95% CI: −4.47 to 3.59)	0.831
Post‐op 1mo	9.00 (IQR 8.00–14.00)^‡^	14.18 ± 5.13^‡^	−3.41 (95% CI: −5.60 to −1.22)	0.002**
Post‐op 6mo	4.55 ± 2.74^‡^ ^§^	5.00 (IQR 3.00–6.00)^‡^ ^§^	0.15 (95% CI: −1.00 to 1.31)	0.795
Post‐op 12mo	2.00 (IQR 1.00–3.00)^‡^ ^§^ ^¶^	1.00 (IQR 0.75–3.00)^‡^ ^§^ ^¶^	0.01 (95% CI: −0.61 to 0.62)	0.987
WOMAC‐stiffness				
Pre‐op	4.00 (IQR 3.00–6.00)	5.00 (IQR 4.00–6.00)	−0.29 (95% CI: −0.93 to 0.35)	0.378
Post‐op 1mo	2.00 (IQR 0.00–2.00)^‡^	3.00 (IQR 2.00–3.00)^‡^	−1.12 (95% CI: −1.61 to −0.62)	< 0.001***
Post‐op 6mo	1.00 (IQR 0.00–3.00)^‡^	2.00 (IQR 1.00–3.00)^‡^ ^§^	−0.24 (95% CI: −0.77 to 0.29)	0.376
Post‐op 12mo	1.00 (IQR 0.00–2.00)^‡^	1.00 (IQR 0.00–2.00)^‡^ ^§^ ^¶^	−0.30 (95% CI: −0.77 to 0.17)	0.208
FJS‐12				
Post‐op 1mo	47.11 ± 9.90	29.39 ± 13.21	17.72 (95% CI: 12.34 to 23.10)	< 0.001***
Post‐op 6mo	67.14 ± 10.74^§^	68.31 ± 10.98^§^	−1.18 (95% CI: −6.25 to 3.89)	0.650
Post‐op 12mo	81.72 ± 6.45^§^ ^¶^	82.73 ± 8.30^§^ ^¶^	−1.01 (95% CI: −4.44 to 2.42)	0.564

* *p* < 0.05.

** *p* < 0.01.

*** *p* < 0.001.

^†^ The GEEs were used to analyze longitudinal trajectory in perioperative CK, CRP, VASs, HHS, WOMAC, and FJS‐12 in both groups. All *p*‐values were adjusted for multiple comparisons using the Bonferroni correction. *p* < 0.05 was considered statistically significant.

^‡^ Compared with the preoperative baseline, the difference was statistically significant, *p* < 0.05.

^§^ Compared with the first follow‐up, the difference was statistically significant, *p* < 0.05.

^¶^ Compared with the second follow‐up, the difference was statistically significant, *p* < 0.05.

^#^ Mean difference (Δ) between RA group and M group.

**FIGURE 3:**
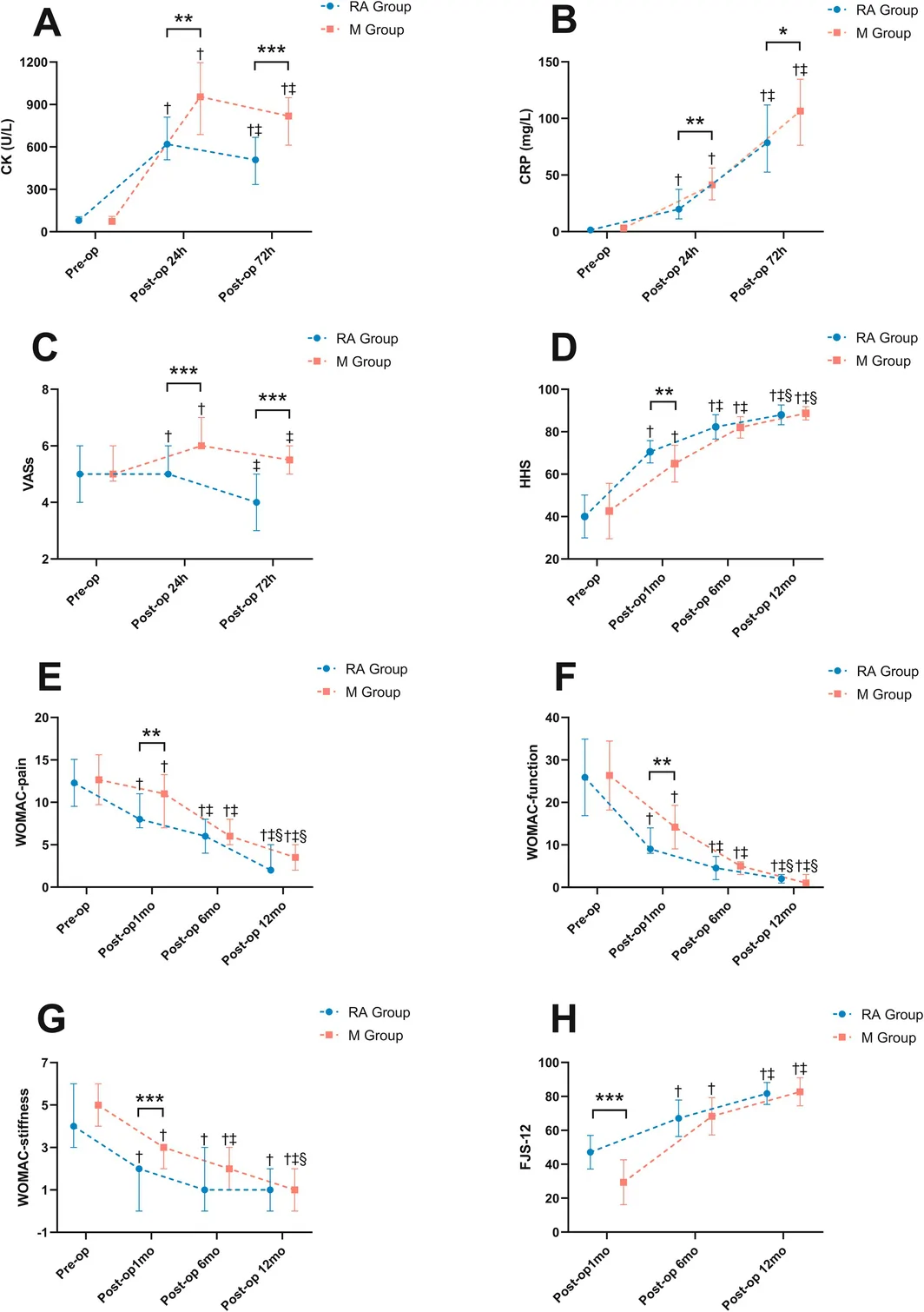
Longitudinal trajectory in perioperative CK, CRP, VASs, HHS, WOMAC, and FJS‐12 in both groups. (A) Longitudinal trajectory in CK. (B) Longitudinal trajectory in CRP. (C) Longitudinal trajectory in VASs. (D) Longitudinal trajectory in HHS. (E) Longitudinal trajectory in WOMAC‐pain. (F) Longitudinal trajectory in WOMAC‐function. (G) Longitudinal trajectory in WOMAC‐stiffness. (H) Longitudinal trajectory in FJS‐12. *p < 0.05. **p < 0.01. ***p < 0.001. ^†^Compared with the preoperative baseline, the difference was statistically significant, *p* < 0.05. ^‡^Compared with the first follow‐up, the difference was statistically significant, *p* < 0.05. ^§^Compared with the second follow‐up, the difference was statistically significant, *p* < 0.05.

Simple effects analysis of group differences revealed: (i) Preoperative baseline of CK, CRP, and VASs did not differ significantly between groups (all *p* > 0.05). However, RA group shown lower CK at Post‐op 24 h (mean difference [Δ] = −300.81; 95% CI: −472.53 to −129.09, *p* = 0.001) and Post‐op 72 h (Δ = −295.78; 95% CI: −428.45 to −163.11, *p* < 0.001) compared to M group. Similarly, RA group exhibited significantly lower VASs at both Post‐op 24 h (Δ = −0.86; 95% CI: −1.33 to −0.38, *p* < 0.001) and Post‐op 72 h (Δ = −1.12; 95% CI: −1.65 to −0.60, *p* < 0.001) compared to M group. Besides, a significant reduction in CRP was observed in RA group relative to M group at both Post‐op 24 h (Δ = −15.71; 95% CI: −26.49 to −4.93, *p* = 0.004) and Post‐op 72 h (Δ = −22.25; 95% CI: −41.91 to −2.59, *p* = 0.027) (Table 3, Figure 3A–C). (ii) At Post‐op 1mo, RA group exhibited significantly superior HHS, WOMAC‐pain, WOMAC‐function, WOMAC‐stiffness, and FJS‐12 (all *p* < 0.01). Conversely, no intergroup differences in patient‐reported outcome measures (PROMs) were observed at Post‐op 6mo and Post‐op 12mo (all *p* > 0.05) (Table 3, Figure 3D–H).

In summary, the key findings demonstrated: (i) The CK and VASs in both groups peaked at Post‐op 24 h, while CRP levels continued to increase throughout the initial 72 h postoperatively. However, the RA group maintained significantly lower CK, CRP, and VASs throughout the initial 72 h postoperatively compared to the M group. (ii) While both groups exhibited statistically significant longitudinal improvements in PROMs, the RA group demonstrated transient superiority at the 1‐month follow‐up assessment. Although this functional advantage was no longer statistically significant at subsequent 6‐ and 12‐month follow‐ups.

### 3.3 Correlation Between TFL Injury and Clinical Outcomes

Spearman correlation analysis revealed that TFL injury index was significantly associated with early adverse clinical outcomes. It showed strong positive correlations with unsupported ambulation duration (*ρ* = 0.775), CK at Post‐op 72 h (*ρ* = 0.619), CRP at Post‐op 72 h (*ρ* = 0.628), and VASs at Post‐op 72 h (*ρ* = 0.652) (all *p* < 0.001). More importantly, TFL injury index demonstrated a strong negative correlation with HHS (*ρ* = −0.615) and FJS‐12 (*ρ* = −0.759) at Post‐op 1mo, while conversely showing moderate positive correlations with WOMAC‐pain (*ρ* = 0.410) and WOMAC‐function (*ρ* = 0.583) at Post‐op 1mo (all *p* < 0.001). This analysis indicates that more severe TFL injury is associated with greater early postoperative pain and inflammatory responses, as well as poorer functional outcomes (Table 4).

**TABLE 4:** Spearman correlation analysis between TFL injury index and clinical outcomes.

Outcome variable	Spearman's *ρ*	*p*	Interpretation
Operative duration	0.054	0.659	None
Duration of hospitalization	−0.017	0.887	None
Perioperative blood loss	0.208	0.087	None
Allogeneic transfusion	0.195	0.109	None
Unsupported ambulation duration	0.775	< 0.001***	Strong positive
CK			
Pre‐op	0.037	0.765	None
Post‐op 24h	0.705	< 0.001***	Strong positive
Post‐op 72h	0.619	< 0.001***	Strong positive
CRP			
Pre‐op	0.076	0.536	None
Post‐op 24h	0.177	0.145	None
Post‐op 72h	0.628	< 0.001***	Strong positive
VASs			
Pre‐op	0.050	0.683	None
Post‐op 24h	0.275	0.022*	Weak positive
Post‐op 72h	0.652	< 0.001***	Strong positive
HHS			
Pre‐op	−0.002	0.984	None
Post‐op 1mo	−0.615	< 0.001***	Strong negative
Post‐op 6mo	−0.076	0.534	None
Post‐op 12mo	−0.100	0.415	None
WOMAC‐pain			
Pre‐op	−0.047	0.698	None
Post‐op 1mo	0.410	< 0.001***	Moderate positive
Post‐op 6mo	0.124	0.310	None
Post‐op 12mo	0.253	0.036*	None
WOMAC‐function			
Pre‐op	0.030	0.808	None
Post‐op 1mo	0.583	< 0.001***	Moderate positive
Post‐op 6mo	−0.025	0.840	None
Post‐op 12mo	0.115	0.345	None
WOMAC‐stiffness			
Pre‐op	0.057	0.642	None
Post‐op 1mo	0.176	0.147	None
Post‐op 6mo	0.135	0.270	None
Post‐op 12mo	0.264	0.028*	Weak positive
FJS‐12			
Post‐op 1mo	−0.759	< 0.001***	Strong negative
Post‐op 6mo	−0.022	0.856	None
Post‐op 12mo	−0.006	0.964	None

* *p* < 0.05.

*** *p* < 0.001.

### 3.4 Radiologic Measurements

The acetabular abduction angle was significantly smaller in RA group (39.89° ± 5.01° vs. 42.82° ± 5.96°, *p* = 0.033) compared to M group while no intergroup difference was observed in the acetabular anteversion angle (*p* > 0.05). Furthermore, RA group demonstrated significantly higher proportions of acetabular components within the Lewinnek safe zone for both abduction (100% vs. 84.2%, *p* = 0.029) and anteversion (100% vs. 81.6%, *p* = 0.014). No significant intergroup differences in LLD, FOD, AOD, or GOD (all *p* > 0.05) (Table 5, Figure 4).

**TABLE 5:** Radiologic measurements in both groups.

	RA group (*n* = 31)	M group (*n* = 38)	Statistic values	*p*
Abduction angle (°)	39.89 ± 5.01	42.82 ± 5.96	*t* = 2.180	0.033*
Safe zone of abduction angle (*n*)				0.029* ^†^
In	31 (100.0%)	32 (84.2%)		
Out	0 (0.0%)	6 (15.8%)		
Anteversion angle (°)	14.08 ± 3.75	14.41 ± 6.00	*t* = 0.267	0.791
Safe zone of anteversion angle (*n*)				0.014* ^†^
In	31 (100.0%)	31 (81.6%)		
Out	0 (0.0%)	7 (18.4%)		
LLD (mm)	1.90 ± 5.16	0.19 (IQR −3.09 to 6.46)	*Z* = −0.911	0.362
FOD (mm)	0.77 ± 6.93	3.02 ± 8.16	*t* = 1.221	0.226
AOD (mm)	−3.56 ± 5.11	−6.14 ± 5.65	*t* = −1.971	0.053
GOD (mm)	−2.79 ± 7.81	−3.12 ± 8.26	*t* = −0.167	0.868

* *p* < 0.05.

^†^ Fisher's exact test.

**FIGURE 4:**
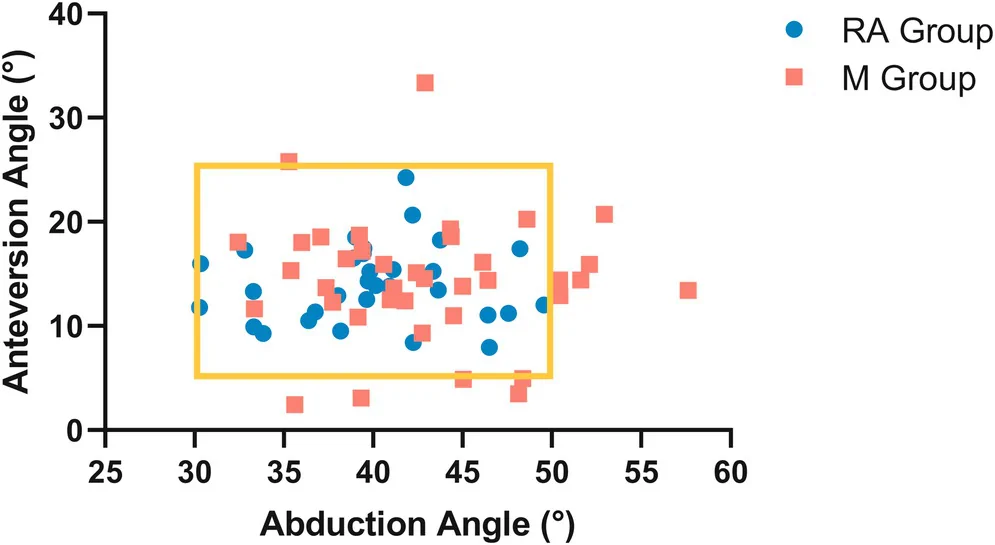
Scatterplot of acetabular components alignment in both groups. Square box reflects the Lewinnek safe zone.

### 3.5 Complications

The incidence of incision‐related adverse events was significantly lower in RA group compared to M group (6/31, 19.4% vs. 20/38, 52.6%, *p* = 0.006, Fisher's exact test). Among specific incision‐related events, incision pain occurred in 3 patients (9.7%) in RA group and 12 patients (31.6%) in M group (*p* = 0.040); incision edema was observed in 0 (0.0%) versus 4 (10.5%) (*p* = 0.122); and abnormal healing in 2 (6.5%) versus 0 (0.0%) (*p* = 0.198). Deep venous thrombosis was noted in 1 patient (3.2%) in RA group and 4 patients (10.5%) in M group (*p* = 0.370). No instances of surgical site infection, periprosthetic joint infection, dislocation, aseptic loosening, reoperations, or readmissions occurred in either group during 12‐month follow‐up.

## 4 Discussion

As previously mentioned, increased soft‐tissue envelope thickness in obese patients compromises surgical exposure of the acetabulum and proximal femur, rendering adequate osseous visualization technically challenging and increasing the risk of trauma to periarticular muscles. Robotic‐arm assisted technology utilizes preoperative CT‐based 3D planning and intraoperative robotic‐arm execution with real‐time haptic feedback; this technology enables precise components implantation within predefined safety boundary. Although substantial evidence confirmed superiority of RA technology in acetabular components positioning accuracy and limb length optimization [18, 31, 32, 33, 34]. No comprehensive analysis had evaluated the efficacy and safety profile of robotic‐arm assisted DAA‐THA for enhancing periarticular muscles preservation and subsequent functional recovery in obese population.

The main findings of this study demonstrate that RA technology significantly facilitates TFL preservation in obese patients undergoing DAA‐THA compared to manual methods. This muscle‐sparing effect correlates with milder systemic inflammation, reduced pain, and accelerated early functional recovery, thus offering superior early clinical outcomes through improved TFL protection.

### 4.1 Robotic‐Arm Assisted DAA‐THA Facilitates TFL Preservation and Early Functional Recovery in Obese Patients

It is well‐established that elevated BMI is associated with increased technical difficulty during THA. The increased soft tissue thickness around the hip in obese patients necessitates more frequent use of retractors and other ancillary instruments to achieve adequate exposure of the acetabulum and proximal femur. Emerging evidence [23, 35, 36] suggests that DAA may exacerbate iatrogenic muscle injury due to excessive traction or contusion in obese patients undergoing THA. While Babar et al. [20] reported that RA technology reduces iatrogenic injury risk by confining surgical instrumentation within the preoperatively surgical plan, thus mitigating aberrant tissue tension and shear forces around the hip.

To comprehensively evaluate the role of contemporary RA technology in periarticular muscle preservation, a comparative analysis was performed on both general and morphological indicators of muscle injury between groups. Serum CK levels had been reported as an objective method for quantifying surgery‐related muscle injury [37]. Moreover, prior research had established an association between the magnitude and duration of the postoperative CK peak and the severity of muscle injury [38]. Therefore, CK was selected as a general indicator to reflect the overall muscle injury associated with the two surgical techniques in this study. According to GEEs' simple effects analysis, the postoperative CK peak occurred at Post‐op 24 h in both groups. However, the RA group demonstrated significantly lower CK at Post‐op 24 h (Δ = −300.81; 95% CI: −472.53 to −129.09) and Post‐op 72 h (Δ = −295.78; 95% CI: −428.45 to −163.11) compared to the M group. These findings suggest a significant muscle‐sparing effect of the RA technique.

Rykov et al. [14] further quantified approach‐specific myotrauma which suggested that posterior approaches predominantly affect external rotators, while DAA impacts TFL and iliopsoas. Frye et al. [15] specifically documented amplified TFL injury in obese patients undergoing DAA‐THA. Therefore, we selected TFL as a validated sentinel indicator of periarticular muscle injury based on prior evidence. This study established a new objective and continuous morphological index, the TFL injury index. Validation confirmed that it is a reliable and valid metric, supporting its adoption as an effective measure of TFL injury. We found that intraoperative TFL injury index was significantly lower in RA group (18.05 [IQR 14.51–30.05] %) compared to M group (38.31 [IQR 20.58–50.70] %). The *E*‐value analysis further supports the robustness of our findings. The observed *E*‐value of 14.3 indicates that an unmeasured confounder would need to be unrealistically strongly associated with both the exposure and the outcome to eliminate the observed effect. Even the lower confidence limit (*E*‐value = 5.3) suggests that a strong unmeasured confounder would be required, which is unlikely in our study. While Spearman's correlation analysis revealed that TFL injury index was strongly positively correlated with postoperative CK levels. Through a systematic comparison of both general and morphological indicators of muscle injury, we suggested that in obese patients, RA technology conferred more effective preservation of periarticular muscles, particularly TFL, compared to manual technique. Perazzini et al. [18] and Xu et al. [19] found that RA technology enables precise bone preparation and prosthesis placement within a confined operative space without extensive soft tissue dissection and retraction. We proposed that, owing to stereotactic haptic feedback and predefined safety boundaries intrinsic to the robotic arm, the surgeon could accurately execute the procedure guided by the system's visual prompts and arm feedback without the need for direct visualization. This approach reduced the need for extensive tissue exposure and retraction, which objectively lessened muscular injury.

### 4.2 Correlation of TFL Injury With Postoperative Inflammation, Pain, and Early Functional Recovery

Early postoperative pain management and functional recovery are critical determinants of optimizing patients' quality of life. Wilson et al. [5] demonstrated that obese patients undergoing THA exhibit increased opioid dependence, assistive device utilization, extended hospital stays, and higher rehabilitation facility transfer rates. In DAA‐THA, the preservation of periarticular muscles is closely associated with postoperative pain levels, rehabilitation duration, and early gait function. This study found that the TFL injury index demonstrated strong positive correlations with CRP (*ρ* = 0.628) and VASs (*ρ* = 0.652) at Post‐op 72 h. It might be due to the surgical dissection of periarticular structures and capsulotomy inciting inflammatory cascades driving early postoperative pain [39]. While RA group exhibited significantly lower CRP and VAS levels within the initial 72 h postoperatively compared to M group. This is consistent with its tissue‐preserving effect, whereby reduced TFL injury manifests as a dampened systemic inflammatory response, translating into clinically lower pain levels.

As a key component of the myofascial kinetic chain, TFL contributes crucially to hip flexion, internal rotation, and abduction. Rosenlund et al. [40] established that preserved hip abductor and flexor strength correlates with optimal early gait quality. Similarly, in this study, we found that TFL injury was significantly associated with early adverse clinical outcomes. Specifically, TFL injury index demonstrated a strong positive correlation with unsupported ambulation duration (*ρ* = 0.775), and patients in RA group achieved unsupported ambulation in a significantly shorter time. More importantly, TFL injury index demonstrated a strong negative correlation with HHS (*ρ* = −0.615) and FJS‐12 (*ρ* = −0.759) at Post‐op 1mo. Conversely, it showed moderate positive correlations with WOMAC‐pain (*ρ* = 0.410) and WOMAC‐function (*ρ* = 0.583) at Post‐op 1mo. Besides, RA group demonstrated significantly superior PROMs at Post‐op 1mo compared to M group, which aligns with prior study results. The comprehensive analysis suggested that by preserving TFL, RA technology significantly reduces postoperative inflammation and pain, ultimately contributing to faster early ambulation and functional recovery. This is of considerable clinical significance. Although this functional advantage was no longer statistically significant at subsequent 6 and 12 months evaluations.

### 4.3 Robotic‐Arm Assisted DAA‐THA Improves Acetabular Component Positioning Without Compromising LLD or Offset Restoration

Soderquist et al. [12] demonstrated that manual DAA‐THA resulted in Lewinnek safe zone violations in 13% of patients with BMI < 35 kg/m^2^ versus 33% in those with BMI > 40 kg/m^2^. Conventional manual DAA relies on intraoperative fluoroscopy, osseous landmarks, and surgeon experience, which are susceptible to positional artifacts, radiographic limitations, and visual field constraints, introducing substantial variability. RA technology now provides precise, reproducible intraoperative guidance, effectively mitigating obesity‐related positioning challenges [17, 41]. Substantial evidence confirmed that precise acetabular components positioning is a critical determinant of functional outcomes and implant longevity in THA [11, 42]. Consequently, an analysis of acetabular implant position was performed for both groups. We found that the RA group demonstrated significantly higher proportions of acetabular components positioned within the Lewinnek safe zone for both abduction (100% vs. 84.2%) and anteversion (100% vs. 81.6%). These findings corroborated prior evidence that RA technology optimizes acetabular components positioning in obesity. However, it must be emphasized that the impact of improved acetabular components positioning on functional recovery is likely a long‐term outcome. Although RA group demonstrated superior precision in acetabular positioning compared to M group, the differences in PROMs were primarily observed at 1‐month follow‐up, with functional convergence in subsequent follow‐ups. Despite ongoing debates regarding the predictive value of the safe zone for dislocation risk [43], no cases of prosthesis dislocation, aseptic loosening, or reoperation were observed during the 12‐month follow‐up period. Within the context of this study, we concluded that the enhancement in acetabular positioning precision may have a limited contribution to the improvement of early functional recovery in obese patients, but the long‐term clinical benefit of this precision is still being established.

Postoperative LLD and the reconstruction of hip offset are critical determinants of functional outcomes following THA. This study also evaluated these parameters. Although Zhang et al. [33] suggested that RA technology can significantly reduce LLD following THA. Curiously, no statistically significant intergroup difference in LLD was observed in our cohort. It might be attributed to the supine positioning inherent to DAA in this study, which facilitates intraoperative LLD assessment through fluoroscopic imaging, direct caliper measurement, and anatomical landmark referencing, enabling real‐time correction even with manual technology [3, 44, 45, 46]. In contrast, the study by Zhang et al. [33] was conducted in patients undergoing THA via posterior approach in lateral decubitus position. The constraints inherent to this positioning might impede accurate limb length assessment when using manual technology. Caus et al. [47] demonstrated that intraoperative anteroposterior radiography provides moderate‐precision LLD estimation during DAA‐THA. Lu et al. [48] corroborated that DAA yields superior LLD precision compared to posterior approaches, achieving more accurate leg length equalization and reducing perceived discrepancies. Consequently, the supine positioning in this study likely contributed to the nonsignificant LLD differences between groups. Furthermore, no significant intergroup differences were detected in FOD, AOD, or GOD. Weißenberger et al. [49] and Antoniadis et al. [50] postulated that obesity exerts limited influence on native femoral offset restoration and acetabular rotation center reconstruction during DAA‐THA, potentially explaining the comparable offset parameters observed herein.

### 4.4 Robotic‐Arm Assisted DAA‐THA Reduces Incision‐Related Adverse Events

Obesity was widely recognized as an independent risk factor for complications following DAA‐THA [22, 51, 52]. Previous studies had discovered that obesity independently elevated 30‐day readmission rates [53] and periprosthetic joint infection risk [54]. In our cohort, RA group demonstrated significantly lower incision pain incidences (9.7% vs. 31.6%) compared to M group. However, no significant differences were observed in incision‐related complication subtypes (including incision edema, abnormal healing, and deep venous thrombosis) except for incision pain incidence. Several factors may explain these findings. Firstly, large‐scale retrospective analyses demonstrated that 1% to 2% of obese patients develop superficial wound dehiscence requiring debridement and primary closure after DAA‐THA [55]. When managed appropriately, these complications did not increase revision surgery or periprosthetic joint infection risk [9]. The incidence of incision‐related complications after DAA‐THA remained low, and our modest cohort size (*n* = 69) might lack sufficient statistical power to detect significant intergroup differences. Secondly, obesity was defined per World Health Organization criteria as BMI ≥ 30 kg/m^2^ in this study. Jahng et al. [56] identified BMI cutoffs of 28 kg/m^2^ (AUC = 0.66; 95% CI: 0.59–0.73) for incision‐related complications and 28.2 kg/m^2^ (AUC = 0.75; 95% CI: 0.66–0.87) for reoperations following DAA‐THA via receiver operating characteristic analysis. Conversely, Salmons et al. [57] defined a BMI threshold of 33 kg/m^2^ as the optimal cutoff for predicting incision‐related complications following DAA‐THA. The median BMIs in our cohorts (31.30 kg/m^2^ for RA group and 31.63 kg/m^2^ for M group) approximated these thresholds. This proximity might have objectively reduced incision‐related complication incidence while masking potential intergroup differences in adverse event profiles. Lastly, John et al. [58] reported that scar adhesion and persistent inflammation resulting from periarticular soft tissue injury are associated with chronic postoperative pain. In this study, RA group demonstrated superior intraoperative preservation of TFL, along with lower VASs and CRP levels within the initial 72 h postoperatively. Although the retrospective design of this study precluded a longer‐term assessment of inflammatory response and pain, significant differences were still observed in this limited cohort. We therefore posited that the preservation of TFL is linked to the mitigation of postoperative inflammation and incisional pain, which might account for the disparity in incision pain incidences between groups.

### 4.5 Robotic‐Arm Assisted DAA‐THA Prolongs Operative Duration

Notably, operative duration was significantly prolonged in RA group (164.39 ± 55.07 min) compared to M group (138.08 ± 53.51 min). This temporal increase primarily reflected the requisite technical processes for robotic system deployment, including reference array installation and optical navigation calibration. Crucially, no significant increase was observed in perioperative blood loss or allogeneic transfusion. Moreover, RA technology did not increase the postoperative complication rate. We therefore concluded that the net clinical benefit of RA technology outweighs the risks associated with extended operative duration.

### 4.6 Strengths and Limitations

The strength of this study lies in being the first to demonstrate, through a retrospective cohort design, the efficacy and safety of robotic‐arm assisted DAA‐THA in facilitating TFL preservation and subsequent functional recovery in obese patients. Furthermore, correlation analyses revealed that RA technology, by preserving TFL, significantly reduced postoperative inflammation and pain, ultimately contributing to faster early ambulation and functional recovery. It might provide a viable solution to achieve superior early functional outcomes with reduced surgical injury by mitigating anatomical challenges inherent to the obese population.

This study still had several limitations. Firstly, its retrospective design introduced potential selection bias in cohort allocation, and unmeasured confounding factors along with the lack of randomization may have influenced the interpretation of the results. We intend to validate these findings through a prospective randomized controlled trial with expanded sample size. Secondly, a computer‐assisted morphometric index (TFL injury index) was utilized and validated in this study. However, inherent in the retrospective design was the absence of postoperative magnetic resonance imaging (MRI) for precise evaluation of muscle edema and necrosis. The interpretation of serum biomarkers (CK and CRP) for muscle injury and inflammation in this study might also be confounded by variables like surgical time, anesthesia, and patient physiology. To address this, a future randomized controlled trial incorporating postoperative MRI was planned for a more conclusive assessment of muscle injury. Finally, the single‐surgeon, single‐center, retrospective design of this study might introduce selection bias. Furthermore, caution should be exercised when generalizing these findings to surgeons who are still on the robotic‐assisted surgery learning curve. Multicenter validation studies incorporating diverse robotic platforms and surgeons at varying experience levels remain warranted.

## 5 Conclusion

In obese patients undergoing DAA‐THA, RA technology significantly reduced intraoperative injury to TFL. This muscle preservation effect was significantly associated with attenuated early postoperative inflammation, lower pain levels, and faster functional recovery. The facilitated TFL preservation afforded by RA technology thus leads to better early clinical outcomes. It might provide a viable solution to achieve superior early functional outcomes with reduced surgical injury by mitigating anatomical challenges inherent to the obese population.

## Author Contributions

**Shijie Chen:** resources, formal analysis, validation, investigation, writing – original draft, conceptualization, methodology. **Dasheng Lin:** validation, investigation, supervision, writing – original draft, writing – review and editing, conceptualization, methodology. **Huihuang Chen:** writing – original draft, conceptualization, methodology. **Wentao Lin:** writing – original draft, conceptualization, methodology. **Dongmin Xu:** writing – original draft, conceptualization, methodology. **Eryou Feng:** validation, investigation, supervision, writing – review and editing, conceptualization, methodology.

## Funding

The authors have nothing to report.

## Ethics Statement

This study was conducted in accordance with the Declaration of Helsinki while approved and supervised by the Ethics Committee of our institution (Approval No. 2025KY408), which provided continuous oversight throughout the investigation period. The Ethics Committee granted waiver of consent for this retrospective study.

## Consent

All authors listed meet the authorship criteria according to the latest guidelines of the International Committee of Medical Journal Editors and have been in agreement with the manuscript.

## Conflicts of Interest

The authors declare no conflicts of interest.

## Data Availability

The data that support the findings of this study are available from the corresponding author upon reasonable request.
